# Double-center observational study of minimally invasive sacroiliac joint fusion for sacroiliac joint dysfunction: one-year results

**DOI:** 10.1186/s13018-022-03466-x

**Published:** 2022-12-28

**Authors:** Sem M. M. Hermans, Rob J. H. Knoef, Valérie N. E. Schuermans, Martijn G. M. Schotanus, Jorm M. Nellensteijn, Henk van Santbrink, Inez Curfs, Wouter L. W. van Hemert

**Affiliations:** 1 Department of Orthopaedic Surgery and Traumatology, Zuyderland Medical Center, Heerlen, The Netherlands; 2 Care and Public Health Research Institute (CAPHRI), Maastricht University, Maastricht, The Netherlands; 3 Department of Orthopaedic Surgery, Medical Spectrum Twente, Enschede, The Netherlands; 4 Department of Neurosurgery, Maastricht University Medical Center +, Maastricht, The Netherlands; 5 Department of Neurosurgery, Zuyderland Medical Center, Heerlen, The Netherlands

## Abstract

**Background:**

For a substantial part of patients with chronic low back pain, the origin is located in the sacroiliac joint (SIJ). Minimally invasive sacroiliac joint fusion (MISJF) is increasingly being implemented as a treatment option in SIJ dysfunction. Despite remaining controversy, evidence continues to increase. This study evaluates the clinical results and safety of MISJF in a double-center consecutive case series in patients with SIJ dysfunction over a one-year observation period.

**Methods:**

SIJ complaints were diagnosed after history taking, physical examination and least a 50% reduction of SIJ pain 30–60 min following image-guided injection. Primary outcome measures were patient reported outcome measurements (PROMs), consisting of Visual Analogue Scale (VAS) pain score and EuroQol 5-dimensions 3-levels (EQ-5D-3L). Patients’ perspectives on the effects of surgery were collected through questionnaires. Secondary outcome measures were implant positioning and (serious) adverse events ((S)AE’s).

**Results:**

A total of 29 patients were included. In 44.8% of patients, SIJ dysfunction was of postpartum origin. The mean VAS-pain score improved from 7.83 (± 1.71) to 4.97 (± 2.63) postoperatively (*p* < 0.001). EQ-5D-3L score improved from 0.266 (± 0.129) to 0.499 (± 0.260) postoperatively (*p* < 0.001). Opioid consumption decreased from 44.8 to 24.1% postoperatively (*p* = 0.026). In 13.7% of patients, an (S)AE occurred.

**Conclusion:**

MISJF appears to be an effective and safe procedure in this cohort. Statistically significant and clinically relevant improvements in pain and quality of life were observed one-year postoperatively. Future studies should focus on the long-term outcomes to further evaluate the safety and effectiveness of MISJF.

**Supplementary Information:**

The online version contains supplementary material available at 10.1186/s13018-022-03466-x.

## Introduction

Chronic low back pain is a common health problem worldwide, and one of the major causes of disability [37]. Sacroiliac joint (SIJ) dysfunction is an often overlooked cause of complaints. In 14–22% of patients with chronic low back pain, the origin is located in the SIJ [1, 4, 29]. The etiology of SIJ dysfunction varies, and most cases include posttraumatic degenerative or postpartum origins [25]. Prior lumbar fusion surgery and connective tissue disorders are also a prevalent risk factor for development of SIJ dysfunction [2, 18]. Despite the available conservative treatment options, many patients continue to have a considerable reduced quality of life (QoL) due to persistent SIJ pain [29]. The impaired QoL for patients with SIJ dysfunction is comparable to other orthopedic conditions with an indication for surgery [6]. Surgical treatment options for SIJ dysfunction have been unattractive for a long period of time. Mainly because open arthrodesis of the SIJ is associated with high morbidity and only moderate long-term results [5, 15, 35]. Currently, several minimally invasive sacroiliac joint fusion (MISJF) systems are available for fusion of the SIJ. Most evidence is obtained with cannulated triangular, titanium implants based on the preponderance of the literature [8, 11, 13, 23, 27, 28]. The initial outcomes of these procedures are promising in terms of pain reduction and improvement of mobility [21]. Despite increasing evidence of effectiveness, controversy remains on the role of interventional procedures, potentially because diagnosing SIJ dysfunction can be elusive or previous open surgery for SIJ pain was not appealing. To this day, there is no recognized standard surgical indication for SIJ dysfunction and often a prolonged conservative trajectory is followed [25]. Most of the current studies on MISJF are industry funded, hence having a potential risk of bias in the reporting of results.

Presently, only a few centers in The Netherlands have introduced MISJF for patients with chronic SIJ dysfunction. This independent study aims to evaluate the results and safety of MISJF in a double-center consecutive case series in patients with SIJ dysfunction over a one-year observation period.

## Material and methods

### Study design

This study was a retrospective study of consecutive series of patients that received MISJF, between April 15, 2019, and June 19, 2020, in two specialized SIJ dysfunction centers in The Netherlands (Zuyderland Medical Center, Heerlen, and Medical Spectrum Twente, Enschede). Patients were selected by chart review, as all patients that underwent MISJF were analyzed. The study outcomes were questionnaire based, with surveys being taken preoperative and one-year postoperative. Preoperative data were collected at the outpatient clinic and postoperative follow-up data was retrieved by contacting patients through mail. Preoperative imaging diagnostics included X-rays and optional computed tomography (CT) scans of the pelvis. A corresponding CT scan was scheduled to be obtained one day following surgery.

### Population

Patients were strictly selected for MISJF based on the following criteria. Prior to surgery, all patients were examined by one of three specialized MISJF surgeons (WvH, IC, and JN). Besides medical interviewing, the examination included the following SIJ provocative tests; flexion abduction external rotation (FABER-test), thigh thrust, Gaenslen’s test, sacral distraction, lateral compression, and sacral thrust [17]. When at least 3 of 5 provocative tests evoked SIJ pain, patients received an image-guided intra-articular SIJ injection with local anesthetic according to a specific guideline [30]. The injections were performed by a specialized MISJF surgeon or experienced pain specialist. Final diagnosis of SIJ dysfunction was based on physical examination and at least a 50% reduction of SIJ pain 30–60 min following fluoroscopy-guided injection with lidocaine 2%. Contrast was used to ensure proper needle placement. Other causes of low back pain were excluded through physical examination and additional imaging, for instance, with spine and/or pelvic radiographs or even through magnetic resonance imaging. All patients had received an extensive conservative treatment trajectory of at least one year, including physical therapy, pelvic compression belt, and SIJ infiltration.

Adult patients who eventually received unilateral or staged bilateral MISJF for SIJ dysfunction were eligible for inclusion. Patients were included when preoperative patient reported outcome measurements (PROMs) and follow-up data, defined as at least one outpatient follow-up visit, were collected and documented in electronic patient records.

### Surgery

All patients were treated with MISJF using a series of triangular titanium, porous titanium plasma spray-coated implants (iFuse Implant System®; SI-BONE, Inc., San Jose, CA, USA). After administration of general anesthesia, the patient was placed in prone position. During MISJF, intraoperative fluoroscopy was used for optimal placement of implants. Lateral view and pelvic inlet and outlet views were utilized to obtain an appropriate starting point. A 3-cm lateral incision was made across the sacral midline. Under lateral fluoroscopy view, the first guide pin was positioned at the appropriate starting point. In- and outlet view was used to place the guide across the ilium and across the SIJ until correct depth was reached. Length of the implant was measured. Subsequently, a drill followed by a triangular broach was used to decorticate the bone and prepare the pathway to receive the first implant. This implant was mostly seated within the sacral ala. Same procedure was repeated for the second and third implant. The second implant was generally located above or adjacent to the S1 foramen and the third between the S1 and S2 foramen. The position and number of implants differed between cases. The incision was then irrigated with bupivacaine, and the tissue layers are sequentially closed.

### Data collection

Data were collected through chart review and stored in a coded and secured database. Besides PROMs, baseline characteristics were collected, which included: sex, age, body mass index (BMI), American Society of Anesthesiologists (ASA) classification, pre- and postoperative use of opioid medication, medical history, medical imaging, surgical technique, (serious) adverse events ((S)AE), and PROMs.

### Follow-up outcomes

The primary outcome measures were PROMs, including Visual Analogue Scale (VAS) pain score (0–10, 10 being “worst pain imaginable”) and EuroQol 5-dimensions 3-levels (EQ-5D-3L, 0.01–1.00, 1.00 indicates “best health state”) and the EQ self-reported health status that records the respondent’s self-rated health (0–100, 100 being “best imaginable health state”). The EQ-5D-3L value was set on “Europe.” Further details on patient’s perspective on the effects of the procedure were evaluated using statements. Possible responses range from strongly agree to strongly disagree, according to the Likert principle [19]. All statements are outlined in the appendix as Additional file 1. The postoperative PROMs questionnaires were mailed to the participants and completed by the patients independently. Secondary outcome measures were opioid consumption, implant positioning on postoperative CT, and (S)AE’s. Musculoskeletal radiologists familiar with MISJF evaluated all CT scans. This evaluation included the position of the implants and the ossification between the sacrum and the ilium on later CT scans. All (S)AE’s, including causes of re-hospitalization, surgical-related events, and reoperations for MISJF, were analyzed as well.

### Statistical analysis

Statistical analyses were carried out using IBM SPSS statistics 27 (Inc., Chicago, IL). All descriptive data are presented as means with standard deviations (SD), frequencies (%), or medians with ranges. Descriptive data were generated for all variables. Univariate analysis was performed for baseline characteristics. Data were tested for normal distribution. When data were normally distributed, a paired t test was used to determine statistical difference between pre- and postoperative data. In case of absence for normal distribution, Wilcoxon signed-rank test was used. Categorical data were assessed using chi-square and Fisher’s exact test. A *p* value ≤ 0.05 was considered statistically significant. EQ-5D-3L index scores were calculated through a European value set [26].

### Ethics, registration, data sharing plan, funding, and potential conflicts of interest

This study has been approved by the Medical Ethical Committee (METCZ20200224) at both participating centers. This study was registered in the Netherlands trial register (registration number: NL9351) and was written in accordance with the STrengthening the Reporting of OBservational studies in Epidemiology (STROBE) guidelines [9].

## Results

### Baseline characteristics

The medical charts of 57 patients that underwent primary MISJF were reviewed, of whom 29 patients were included. In these 29 patients, pre- and postoperative data were available. Baseline characteristics are presented in Table 1. The majority of patients in this cohort were women (86.2%) with a mean age of 45.6 years. In most cases, the cause of SIJ dysfunction was of postpartum origin (44.8%), followed by Ehlers–Danlos syndrome (EDS) (13.8%). In the first year of follow-up, six (20.7%) patients underwent a staged bilateral procedure. In 7 patients (24.1%), degenerative changes to the SIJ were observed (e.g., vacuum phenomena or sclerosis of the endplates) on preoperative imaging. There were no patients with sacral dysmorphism in this cohort. Almost all patients received three implants over the SIJ during surgery (93.1%). The average procedure duration was 47.8 min (± 14.7). Further characteristics regarding the index procedure are outlined in Table 2.

**Table 1:** Baseline characteristics

Characteristics	Value
Age, years	45.6 (± 8.6)
Women	25 (86.2%)
BMI, kg/m^2^	27.1 (± 4.4)
ASA	
I	5 (17.2%)
II	22 (75.9%)
III	2 (6.9%)
Preoperative opioid consumption	13 (44.8%)
Medical history	
Prior spinal fusion	3 (10.3%)
Prior MISJF (other side)	4 (13.8%)
Other spine surgery	3 (10.3%)
Preoperative imaging abnormality	
None	13 (44.8%)
Degenerative SIJ	7 (24.1%)
Other	9 (30.9%)
Cause of SIJ dysfunction	
Postpartum	13 (44.8%)
Prior spinal fusion	3 (10.3%)
Ehlers–Danlos syndrome	4 (13.8%)
Posttraumatic	2 (6.9%)
Degenerative	2 (6.9%)
Unknown	5 (17.2%)

Data are presented as frequency (n, %) or mean (range)

**Table 2:** Index procedure characteristics

Characteristics	Value
Side, right	16 (55.2%)
Amounts of implants placed	
2	2 (6.9%)
3	24 (93.1%)
Procedure duration, minutes	47.8 (± 14.7)
Adverse events	
Intraoperative	1 (3.4%)
Postoperative	3 (10.3%)
Loosening of implants	2 (6.8%)
Wound infection	1 (3.4%)

Data are presented as frequency (n, %) or mean (range)

### Primary outcome measures

A statistically significant reduction in pain occurred at one-year following surgery compared to baseline (*p* < 0.001). Mean VAS-pain score improved from 7.83 (± 1.71) pre- to 4.97 (± 2.63) at one-year postoperative with a mean change of 2.86 (± 2.94) points. In nine patients (30.9%), a VAS-pain score of 3 or lower was reported. QoL measured through EQ-5D-3L revealed a statistically significant mean improvement of 0.232 (± 0.243) points (*p* < 0.001). The VAS on self-reported health status also improved with statistical significance by 11.7 (± 28.3) points following surgery (*p* = 0.035). Complete data regarding VAS-pain and EQ-5D-3L outcomes are outlined in Table 3.

**Table 3:** Results

Outcome	Preoperative	1 year postoperative	Mean difference	*p*-value
Pain, VAS	7.83 ± 1.71	4.97 ± 2.63	2.86 ± 2.94	< 0.001
Quality of life, EQ-5D-3L	0.266 ± 0.129	0.499 ± 0.260	0.232 ± 0.243	< 0.001
Self-reported health status, VAS	49.6 ± 19.8	61.2 ± 21.4	11.7 ± 28.3	0.035

All values are mean ± SD, and *p* value refers to paired *t* test

**Fig. 1:**
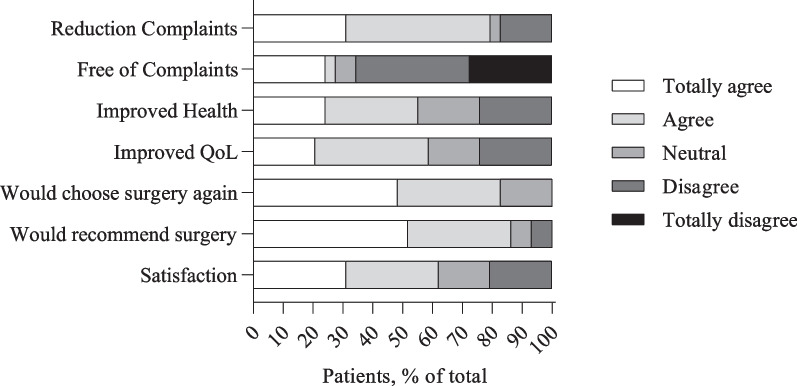
Patient’s perspective on the effects of procedure

Twenty-three patients (79.3%) “agree” or “totally agree” on the statement whether their complaints reduced following surgery. Eight patients (27.5%) “agree” or “totally agree” to be completely free of complaints after treatment. When looking at improved health or QoL, we observe an almost similar response. Health improved in 16 patients (55.2%) following treatment and 17 patients (58.6%) “agree” or “totally agree” that their QoL improved. When asked if patients would have the same surgery for the same result again, 24 patients (82.8%) “agree” or “totally agree.” Twenty-five patients would recommend the same surgery to individuals with similar complaints (86.2%). Finally, 18 patients (62.1%) are satisfied with the results of the procedure. Results of the statements regarding patient’s perspective on effects of the procedure are displayed in Fig. 1.

### Secondary outcome measures

Thirteen patients (44.8%) consumed opioids preoperatively. At one-year postoperatively, this number decreased to seven patients (24.1%). This difference reached statistical significance (*p* = 0.026).

**Fig. 2:**
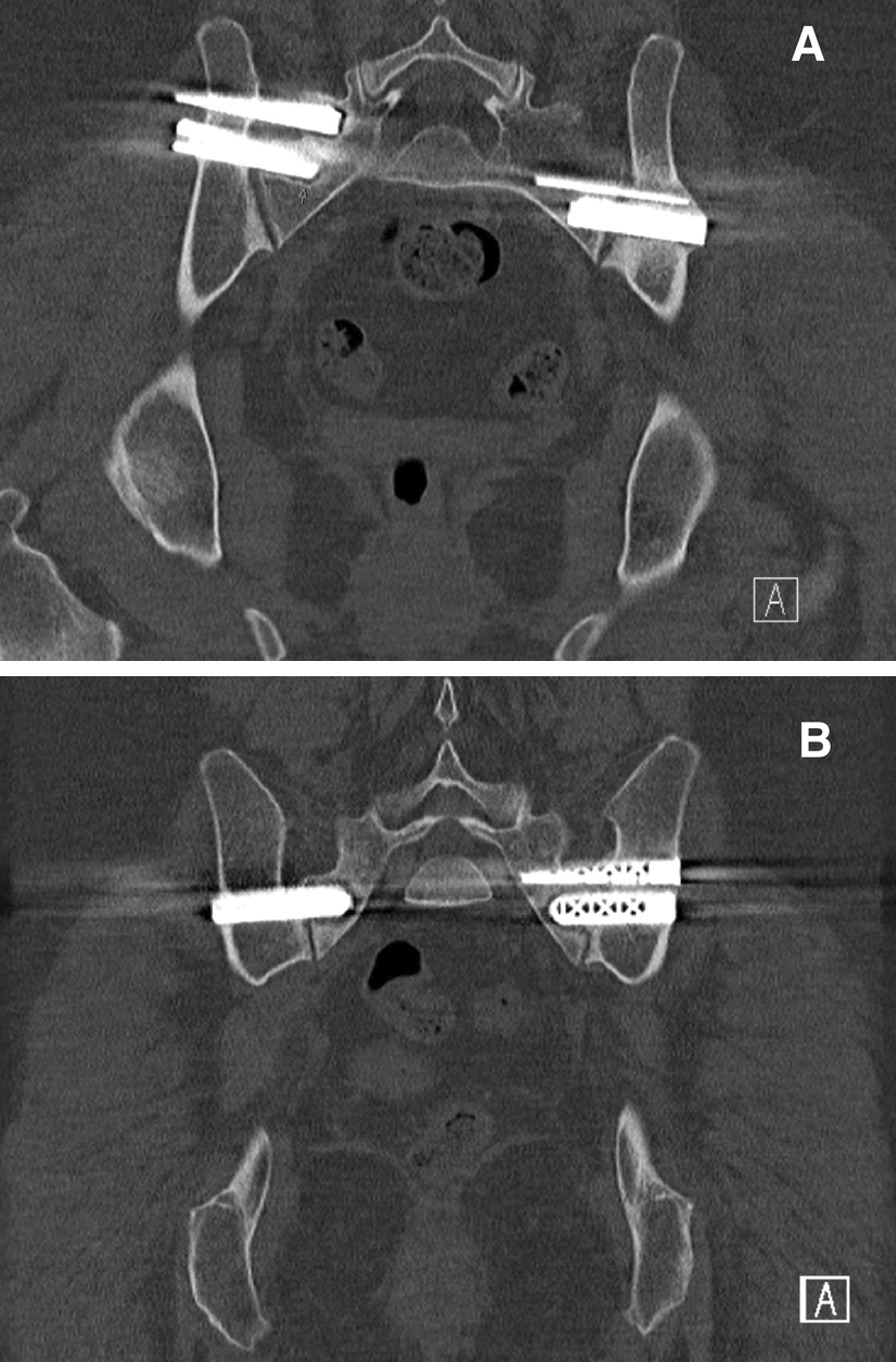
Two cases of implant loosening on pelvic CT scan

Four adverse events occurred: one nerve root injury, one surgical wound infection, and two cases of implant loosening. All except one patient (N = 28) received a pelvic CT scan on the first postoperative day. In 27 of 28 patients (96%), the CT scan revealed adequate positioning of implants. The patient with nerve root injury developed complaints of radiating pain and mild paresthesia in the right leg directly after surgery. The CT scan revealed corresponding nerve root compression of S1, caused by the most cranially located implant. However, no revision surgery was performed and complaints slowly abated during follow-up. The patient with postoperative surgical wound infection reported to the emergency department with wound leakage on the third postoperative day. Debridement surgery was performed, and intravenous antibiotic (AB) therapy was administered. The patient returned home in adequate clinical condition, and AB therapy was concluded for two weeks. The two cases of implant loosening were detected at subsequent CT scans at 6 and 12 months postoperatively. Radiolucency around the affected implants was observed without any intra-articular bridging of trabeculae over the SIJ (Fig. 2). Both patients complained of persisted SIJ pain during follow-up. Revision surgery is planned to revise the loose implants.

In both cases, radiolucency around the implants on the right can be detected without any intra-articular bridging of trabeculae over the SIJ.

## Discussion

This study provides insight on the effectiveness of MISJF in a double-center cohort in a consecutive series of patients with SIJ dysfunction. Overall, we found significant improvements in pain and quality of life, with a low rate of (serious) adverse events, one-year following surgery. We report a mean VAS-pain improvement of 2.86 points and an EQ-5D-3L improvement of 0.232 points. These PROMs are accompanied with an overall satisfaction rate of 62.1%. These results are less effective when compared to some studies in the existing literature. A lot of studies report a VAS-pain improvement of around 4.5 points and satisfaction rates of around 80% following MISJF [8, 13, 21, 28]. A potential explanation for this is that bilateral SIJ dysfunction is a common entity. Typically, there is one more symptomatic side, and in a few cases MISJF for one side is followed by MISJF on the other side because of remaining complaints. Possibly, some postoperative patients did not show significant improvements in pain and QoL as the other SIJ was still symptomatic and requires surgery as well. In our study, a significant proportion of patients suffer from EDS compared to the existing literature. Patients with EDS often suffer from chronic pain as a major source of disability [20]. The difference in patient population is a potential reason for the higher pain score and lower QoL reported postoperatively. In addition, some of these studies implemented eligibility criteria as a baseline score of at least 30% on the Oswestry Disability Index and a VAS-pain score of at least 50 (0–100 scale). There are some independent studies in which the results are more in line with the findings we report in this study [14, 16].

Baseline characteristics of the present cohort show that SIJ dysfunction mostly affects younger women. In most cases, the cause of SIJ dysfunction was of postpartum origin. These data are in line with previous published studies on SIJ dysfunction [11, 24, 33]. In the present study, a significant number of patients suffer from EDS, as a cause of SIJ dysfunction. Only one case series has been published on MISJF in patients with EDS, with successful outcomes [2]. It would be interesting to see future studies focus on this population. Additional baseline characteristics reveal a high prevalence of patients consuming opioids before surgery, revealing a significant degree of pain in daily life. A recent study by Dengler et al. noted similar opioid consumption in patients with SIJ dysfunction (52.5%). Opioid consumption remained the same in the conservatively treated patients (46.9%), while it significantly decreased following MISJF (57.7–44.2%) [12]. For comparison, opioid consumption in patients with knee or hip osteoarthritis is reported to be around 23.6% [31]. Furthermore, individuals with postpartum SIJ pain are often unable to stay active in their workplace [3]. All these findings are in line with the high preoperative VAS-pain score of 7.83, reported in this cohort. The VAS-pain score one-year following surgery was statistically significantly lower. Although the difference seems moderate at first, it reaches the minimal clinically important difference (MCID) according to Kube et al. who defined a reduction of 2.0 points to be clinically relevant [16]. It is recommended that MCID’s should be considered context-specific and take into account the level of pain at baseline [22]. Baseline VAS-pain was high in the presented cohort, which means even modest changes could be of importance. At an individual level, MCID for VAS-pain was reached in 69% of patients. The reported improvement in EQ-5D-3L score also reached MCID [7]. The mean EQ-5D-3L one-year following MISJF was 0.499, which, according to Whynes et al., remains to be a moderate level of daily discomfort [36]. In 72.4% of patients, the MCID for EQ-5D-3L was reached. Conforming EQ-5D-3L score, the remaining VAS-pain score also suggests some level of pain still exists in our patients one-year following surgery. However, according to our exploration of patient’s perspective on the effects of the procedure, 62.1% of patients state to be satisfied with the clinical outcome. Even more patients (82.8%) state they would have the same surgery again knowing the outcome. This could partially be psychological, as patients have often been in long-lasting and unsuccessful rehabilitation. Many of our patients have had symptoms for several years before final diagnosis of SIJ dysfunction was established. Throughout this period, patients have often seen countless specialists and were treated inadequately. Therefore, surgery may feel like a last resort to them. Hence, patients expectations and wish for surgery might be increased. This may bias their interpretation of pain reduction following a diagnostic injection, resulting in poorer surgical outcomes. At the same time, patients may perhaps be more forgiving and positive toward the results of MISJF. This might partly explain the discrepancy in satisfaction and the choice to have surgery again. Around one in four patients states to be completely free of complaints one-year following surgery. Therefore, expectation management plays a significant role in the treatment of SIJ dysfunction. Besides surgical treatment, a holistic approach should be considered, including psychological problems [34].

The rate of (S)AE’s in the present study population is in line with prior published studies [11, 24]. Revision surgery is required in two patients where implant loosening occurred, accompanying inadequate fusion of the SIJ. No predisposing patient factors linked to implant loosening could be identified. Both patients underwent staged bilateral MISJF, and implant loosening occurred in the first operated side. Although these patients suffered an AE, they both stated that they would have surgery again. VAS-pain score improved from 8 to 7 and from 6 to 3 points, respectively. These data indicate that some relief of complaints occurred following both surgeries. Revision surgery is planned with the aim to further improve clinical outcome in these patients, as a newly performed diagnostic SIJ injection reduced complaints. We aim to remove the loose implants and place new implants in an additional trajectory. When there is no sufficient additional trajectory available, the new implant will be rotated to ensure proper fixation.

### Limitations

This study is bound by some limitations. First this a retrospective study, in which not all data could be retrieved from the patient charts resulting in exclusions, potentially leading to selection bias based on completeness of PROMs dataset. The COVID-19 pandemic was a major reason for the significant loss of preoperative PROMs, as these were not collected during this period. The sample size may seem small at first, but can be considered adequate as SIJ dysfunction is only diagnosed in few people. Furthermore, a sample size of 29 patients is in line with prior published studies on MISJF [8, 32].

Patient’s perspective on the effects of the procedure was evaluated using a non-validated questionnaire. The other outcome measurements (VAS-pain and EQ-5D-3L) are validated tools. Nonetheless, they remain PROMs and are thereby at risk for some sort of subjective discrepancies. This could explain why the satisfaction rate is lower than the percentage of individuals who would have surgery again for the same results. These differences in numbers feel conflicting.

Finally, the study length of one year is short. It would be interesting to see long-term follow-up of our patients, especially when a relatively large number of patients indicate to have a neutral perspective on the effects of the procedure. Prior studies with longer follow-up showed excellent results up to 6 years following surgery [10, 33].

Despite the above-mentioned limitations, we were able to obtain data that are still insightful for future studies. Presently, only a few studies are available in Europe that describe the effectiveness of MISJF.

## Conclusion

This independent study presents a two-center retrospective cohort of 29 consecutive patients who underwent MISJF for SIJ dysfunction. Although the sample size is limited, MISJF indicates to be a safe and reasonably effective procedure, with acceptable satisfaction rates and significant improvements in pain and QoL reported one-year following surgery. Future studies should focus on the long-term results to further evaluate the effectiveness of MISJF.

## Supplementary Information

**Additional file 1**. Statements on patient’s perspective on the effects of the procedure.

## Data Availability

Not applicable.

## References

[1] Barros G, McGrath L, Gelfenbeyn M (2019). Sacroiliac joint dysfunction in patients with low back pain. Fed Pract.

[2] Beijk I, Knoef R, van Vugt A, Verra W, Nellensteijn J (2021). Sacroiliac joint fusion in patients with Ehlers Danlos Syndrome: a case series. N Am Spine Soc J.

[3] Bergström C, Persson M, Mogren I (2016). Sick leave and healthcare utilisation in women reporting pregnancy related low back pain and/or pelvic girdle pain at 14 months postpartum. Chiropr Man Therap.

[4] Bernard TN, Kirkaldy-Willis WH (1987). Recognizing specific characteristics of nonspecific low back pain. Clin Orthop Relat Res.

[5] Buchowski JM, Kebaish KM, Sinkov V, Cohen DB, Sieber AN, Kostuik JP (2005). Functional and radiographic outcome of sacroiliac arthrodesis for the disorders of the sacroiliac joint. Spine J.

[6] Cher D, Polly D, Berven S (2014). Sacroiliac joint pain: burden of disease. Med Devices Evid Res.

[7] Coretti S, Ruggeri M, McNamee P (2014). The minimum clinically important difference for EQ-5D index: a critical review. Expert Rev Pharmacoecon Outcomes Res.

[8] Cummings J, Capobianco RA (2013). Minimally invasive sacroiliac joint fusion: one-year outcomes in 18 patients. Ann Surg Innov Res.

[9] Cuschieri S (2019). The STROBE guidelines. Saudi J Anaesth.

[10] Darr E, Meyer SC, Whang PG, Kovalsky D, Frank C, Lockstadt H, Limoni R, Redmond A, Ploska P, Oh MY, Cher D, Chowdhary A (2018). Long-term prospective outcomes after minimally invasive trans-iliac sacroiliac joint fusion using triangular titanium implants. Med Devices Evid Res.

[11] Dengler J, Kools D, Pflugmacher R, Gasbarrini A, Prestamburgo D, Gaetani P, Cher D, Van Eeckhoven E, Annertz M, Sturesson B (2019). Randomized trial of sacroiliac joint arthrodesis compared with conservative management for chronic low back pain attributed to the sacroiliac joint. J Bone Jt Surg Am.

[12] Dengler J, Sturesson B, Kools D, Prestamburgo D, Cher D, van Eeckhoven E, Erk E, Gasbarrini A, Pflugmacher R, Vajkoczy P (2018). Risk factors for continued opioid use in conservative versus surgical management of low back pain originating from the sacroiliac joint. Glob Spine J.

[13] Duhon BS, Bitan F, Lockstadt H, Kovalsky D, Cher D, Hillen T, Wilhite E, Farris J, Newman C, Pestka L, Tao C, Makowski J, Kelly T, Meyer SC, Jones V, Vogt M, Kutz S, Thompson L, Kondrashov D, Kondrashov I, Redmond AJ, Piazza J, Doredant L, Short B, Mayfield J, Soo CL, White J, Haynes K, Pfister A, Mesiwala A, Bose S, Rudolf L, Thibodeau J, Stevenson K, Mahoney L, Gomez S, Stevenson J, Marichal A, Sachs D, Cambron R, White M, Colburn A, Raiden S, Chowdhary A, Fortney T, Thaiyananthan G, Williams T, Oh M, Schmidt G, Yeager M, Wiles D, Maye S, Hasz M, Califano C, Rosenberg W, McCann P, Coe JD, Coe J, Coe M, Vanichkachorn J, Lynch J, Gillespy MC, Zicker S, Rashbaum R, Rusch S, Darr EA, Glaser JA, Fields L, Baczko M (2015). Triangular titanium implants for minimally invasive sacroiliac joint fusion: 2-year follow-up from a prospective multicenter trial. Int J Spine Surg.

[14] Kancherla VK, McGowan SM, Audley BN, Sokunbi G, Puccio ST (2017). Patient reported outcomes from sacroiliac joint fusion. Asian Spine J.

[15] Kibsgård TJ, Røise O, Stuge B (2014). Pelvic joint fusion in patients with severe pelvic girdle pain: a prospective single-subject research design study. BMC Musculoskelet Disord.

[16] Kube R, Muir J (2016). Sacroiliac joint fusion: one year clinical and radiographic results following minimally invasive sacroiliac joint fusion surgery. Open Orthop J.

[17] Laslett M, Aprill CN, McDonald B, Young SB (2005). Diagnosis of sacroiliac joint pain: validity of individual provocation tests and composites of tests. Man Ther.

[18] Lee YC, Lee R, Harman C (2019). The incidence of new onset sacroiliac joint pain following lumbar fusion. J Spine Surg.

[19] Likert R (1932). A technique for the measurement of attitudes. Arch Psychol.

[20] Malfait F, Colman M, Vroman R, De Wandele I, Rombaut L, Miller RE, Malfait A-M, Syx D (2021). Pain in the Ehlers-Danlos syndromes: mechanisms, models, and challenges. Am J Med Genet C Semin Med Genet.

[21] Martin CT, Haase L, Lender PA, Polly DW (2020). Minimally invasive sacroiliac joint fusion: the current evidence. Int J Spine Surg.

[22] Olsen MF, Bjerre E, Hansen MD, Hilden J, Landler NE, Tendal B, Hróbjartsson A (2017). Pain relief that matters to patients: Systematic review of empirical studies assessing the minimum clinically important difference in acute pain. BMC Med.

[23] Polly DW, Cher DJ, Wine KD, Whang PG, Frank CJ, Harvey CF, Lockstadt H, Glaser JA, Limoni RP, Sembrano JN (2015). Randomized controlled trial of minimally invasive sacroiliac joint fusion using triangular titanium implants vs nonsurgical management for sacroiliac joint dysfunction: 12-month outcomes. Neurosurgery.

[24] Polly DW, Swofford J, Whang PG, Frank CJ, Glaser JA, Limoni RP, Cher DJ, Wine KD, Sembrano JN, INSITE Study Group (2016). Two-year outcomes from a randomized controlled trial of minimally invasive sacroiliac joint fusion vs. non-surgical management for sacroiliac joint dysfunction. Int J Spine Surg.

[25] Raj MA, Ampat G, Varacallo M. Sacroiliac joint pain. StatPearls [Internet]. Treasure Island (FL): StatPearls Publishing; 2022. Treasure Island (FL), 2021. https://www.ncbi.nlm.nih.gov/books/NBK470299/. 29261980

[26] rdrr.io. eq5d: methods for analysing “EQ-5D” data and calculating “EQ-5D” index scores. n.d. https://rdrr.io/cran/eq5d/man/eq5d-package.html.

[27] Rudolf L (2012). Sacroiliac joint arthrodesis-MIS technique with titanium implants: report of the first 50 patients and outcomes. Open Orthop J.

[28] Sachs D, Capobianco R, Cher D, Holt T, Gundanna M, Graven T, Shamie AN, Cummings J (2014). One-year outcomes after minimally invasive sacroiliac joint fusion with a series of triangular implants: a multicenter, patient-level analysis. Med Devices Evid Res.

[29] Sembrano JN, Polly DW (2009). How often is low back pain not coming from the back?. Spine (Phila Pa 1976).

[30] Society ISI, Bogduk N. ISIS practice guidelines for spinal diagnostic and treatment procedures, 2nd ed. International Spine Intervention Society; 2014. https://books.google.nl/books?id=8w6NoAEACAAJ.

[31] Thorlund JB, Turkiewicz A, Prieto-Alhambra D, Englund M (2019). Opioid use in knee or hip osteoarthritis: a region-wide population-based cohort study. Osteoarthr Cartil.

[32] Vanaclocha-Vanaclocha V, Verdú-López F, Sánchez-Pardo M, Gozalbes-Esterelles L, Herrera JM, Rivera-Paz M, Martínez-Gómez D (2014). Minimally invasive sacroiliac joint arthrodesis: experience in a prospective series with 24 patients. J Spine.

[33] Vanaclocha V, Herrera JM, Sáiz-Sapena N, Rivera-Paz M, Verdú-López F (2018). Minimally invasive sacroiliac joint fusion, radiofrequency denervation, and conservative management for sacroiliac joint pain: 6-year comparative case series. Neurosurgery.

[34] Vleeming A, Albert HB, Ostgaard HC, Sturesson B, Stuge B (2008). European guidelines for the diagnosis and treatment of pelvic girdle pain. Eur spine J Off Publ Eur Spine Soc Eur Spinal Deform Soc Eur Sect Cerv Spine Res Soc.

[35] Waisbrod H, Krainick JU, Gerbershagen HU (1987). Sacroiliac joint arthrodesis for chronic lower back pain. Arch Orthop Trauma Surgery.

[36] Whynes DK, McCahon RA, Ravenscroft A, Hodgkinson V, Evley R, Hardman JG (2013). Responsiveness of the EQ-5D health-related quality-of-life instrument in assessing low back pain. Value Health.

[37] Wu A, March L, Zheng X, Huang J, Wang X, Zhao J, Blyth FM, Smith E, Buchbinder R, Hoy D (2020). Global low back pain prevalence and years lived with disability from 1990 to 2017: estimates from the Global Burden of Disease Study 2017. Ann Transl Med.

