# Supplementary material for: Double-center observational study of minimally invasive sacroiliac joint fusion for sacroiliac joint dysfunction: one-year results
Source: J Orthop Surg Res. 2022 Dec 28;17:570. doi: 10.1186/s13018-022-03466-x (PMC9794474; doi:10.1186/s13018-022-03466-x)
Supplement: Supplementary file 1 — Additional file 1. Statements on patient’s perspective on the effects of the procedure. [file 13018_2022_3466_MOESM1_ESM.docx]

**APPENDIX**

**Additional file 1: Statements on patient’s perspective on the effects of the procedure**

******
